# Rabies in the Americas: 1998-2014

**DOI:** 10.1371/journal.pntd.0006271

**Published:** 2018-03-20

**Authors:** Mary Freire de Carvalho, Marco A. N. Vigilato, Julio A. Pompei, Felipe Rocha, Alexandra Vokaty, Baldomero Molina-Flores, Ottorino Cosivi, Victor J. Del Rio Vilas

**Affiliations:** 1 Zoonosis group, Pan American Foot-and-Mouth Disease Center (PANAFTOSA), Pan American Health Organization (PAHO/WHO), Rio de Janeiro, Brazil; 2 School of Veterinary Medicine, University of Surrey, Guildford, United Kingdom; Wistar Institute, UNITED STATES

## Abstract

Through national efforts and regional cooperation under the umbrella of the Regional Program for the Elimination of Rabies, dog and human rabies have decreased significantly in Latin America and Caribbean (LAC) countries over the last three decades. To achieve this decline, LAC countries had to develop national plans, and consolidate capabilities such as regular mass dog vaccination, opportune post-exposure prophylaxis and sensitive surveillance. This paper presents longitudinal data for 21 LAC countries on dog vaccination, PEP and rabies surveillance collected from the biannual regional meeting for rabies directors from 1998–2014 and from the Regional Epidemiologic Surveillance System for Rabies (SIRVERA). Differences in human and dog rabies incidence rates and dog vaccination rates were shown between low, middle and high-income countries. At the peak, over 50 million dogs were vaccinated annually in national campaigns in the countries represented. The reported number of animal exposures remained fairly stable during the study period with an incidence rate ranging from 123 to 191 reported exposures per 100,000 people. On average, over 2 million doses of human vaccine were applied annually. In the most recent survey, only 37% of countries reported that they had sufficient financial resources to meet the program objectives. The data show a sufficient and sustained effort of the LAC countries in the area of dog vaccination and provide understanding of the baseline effort required to reduce dog-mediated rabies incidence.

## Author summary

Globally, dog-mediated rabies kills over 55,000 people on an annual basis and disproportionally affects socio-economically disadvantaged populations. For over 4 decades, the Latin America and Caribbean (LAC) region have worked towards the elimination of dog-mediated rabies succeeding in a substantial reduction of both human and dog rabies cases. This paper summarizes the last 15 years of the national and regional rabies elimination efforts, through a series of performance indicators, including government mass dog vaccination campaigns and post-exposure prophylaxis for humans. The most important message from the tail of the rabies epidemic in the LAC Region is one of endurance by the countries and the region to achieve rabies control and elimination. The performance indicators shown in this paper are tools for the LAC region to evaluate its progress and refine its approach, and for other regions to understand the scope of the effort required to reach elimination.

## Introduction

Dog-mediated rabies still poses a heavy burden on the most disadvantaged populations in the world [1]. In Latin America and Caribbean (LAC) countries, dog and human rabies have decreased significantly over the last three decades due to interruption of rabies virus circulation between dogs and consequently, dog-mediated transmission to humans [2,3]. This success has come from national efforts and regional cooperation under the umbrella of the Regional Program for the Elimination of Rabies. The commitment from PAHO member states to eliminate rabies was first discussed in 1983 during the Meeting of the Directors of National Programs for Rabies in the Americas (REDIPRA), and was then endorsed by the American Meeting at Ministerial Level on Health and Agriculture (RIMSA) [4]. While many countries had rabies control programs prior to the beginning of the regional effort, the program has served as a coalescing force for the region. Since the beginning of the regional elimination effort in 1983, the burden of dog-mediated rabies in LAC countries has decreased by 95% with only six countries reporting dog-mediated human cases in 2015, and three in 2016 [5].

To achieve this decline, LAC countries had to develop and deploy national capabilities as part of comprehensive rabies control and elimination programmes, namely regulation on compulsory notification of rabies cases in humans and animals, surveillance related sample and data flows, national plans properly budgeted and resourced, dog vaccination, and freely available post-exposure prophylaxis (PEP). Data was generated on their performance which, aggregated, can be used to illustrate the Region’s efforts towards the control and elimination of the disease. Regular aggregation of rabies data at the regional level is mostly case-centric, via regular reporting of cases by the countries to the Regional Epidemiologic Surveillance System for Rabies (SIRVERA), maintained by the Veterinary Public Health area of PAHO [2,6]. Aware of the importance of not just registering cases, but also measuring capacities, PAHO developed regular surveys to the countries, prior to the regular REDIPRA meetings, to capture this information.

The World Health Organization (WHO) has targeted dog-transmitted rabies for global elimination by 2030 [7]. Reaching zero human cases and breaking rabies transmission in dogs will be the consequence of a sufficient, efficacious and sustained implementation of rabies control measures. Using longitudinal data on rabies program capacities and case data, this paper provides an understanding of the historical course of the program and allows for evaluation of the continued regional actions towards the final goal of eliminating dog-mediated human rabies. As a forerunner in the dog rabies elimination efforts, the Americas can impart valuable knowledge on the intensive and sustained efforts required for reaching regional elimination.

## Materials and methods

### REDIPRA data

Since 1998, prior to the regular REDIPRA meetings coordinated by PAHO and attended by health and agricultural officials from most of the countries in the Americas, PAHO distributes a survey to all rabies program managers to collect data on the status of their rabies programs capacities and supporting functions (e.g. personnel, resources). These capacities include PEP, human and dog surveillance, and dog vaccination among others [8].

Data from the eight REDIPRA (from REDIPRA V to REDIPRA XV) questionnaires collected from 1998 to 2014 were collated to create a longitudinal dataset. The following twenty-one countries, categorized by their 2014 World Bank Lending Groups (GNI per capita) [9] to facilitate comparisons, contributed data throughout the period: low and low-middle income—Bolivia, El Salvador, Guatemala, Haiti, Honduras and Nicaragua–; upper middle income—Belize, Brazil, Costa Rica, Cuba, Colombia, Dominican Republic, Ecuador, Panama, Paraguay, Peru and Mexico–; and High Income—Argentina, Chile, Uruguay and Venezuela.

The REDIPRA questionnaires varied widely in content and granularity. Consistently, country data on dog population, vaccine type (Nerve Tissue Vaccines (NTV) vs. cell-based vaccine), vaccine coverage of the dog population, number of animal exposures and PEP usage were collected. Sporadically, data on rabies program budgets and expenditures and human resources were also collected.

Data on rabies-immunoglobulin (RIG), and vaccine procurement (costs and mechanisms) was sporadic and of poor quality prior to the REDIPRA XV questionnaire. Likewise, only the last questionnaire captured data on the number of dogs sampled for rabies surveillance. Therefore, only 2013–2014 data from the REDIPRA XV questionnaire for these three capacities is presented in the paper. In REDIPRA XV, Trinidad and Tobago participated in the survey for the first time, while three of the 21 countries included in the longitudinal data (1998–2014) did not participate; therefore, for those analyses that are limited to the final survey (2013–2014) a total of 19 countries are considered.

### SIRVERA data

To complement the REDIPRA data, human and animal rabies case data from 1983 to 2014, including type (whether it was on clinical and epidemiological grounds, laboratory-based or unspecified), PEP status of the human cases, and the aggressor animal for human cases were extracted from the regional rabies database “SIRVERA” managed by PAHO. For the purposes of this paper, cases were considered dog-mediated when the aggressor animal was reported as dog as no variant data is available longitudinally.

### World Bank data

Annual population estimates for the study countries [10] and 2014 World Bank Lending Groups (GNI per capita) [9] were downloaded from the World Bank website.

### Analysis

Human and dog rabies incidences were estimated. The denominator for human rabies incidence was the countries’ annual human population from the World Bank [10]. The dog population estimates reported by the countries in the REDIPRA questionnaires were used for dog rabies incidence. To enable comparisons across time, the incidence of animal exposures reported per 100,000 persons and the ratio of PEP doses to animal exposures was also calculated.

To estimate the dog vaccine coverage, the number of canine doses applied over the estimated dog population was used. Three countries in 2013 and 2014 provided the number of dogs vaccinated, but not the number of doses applied; in these cases, the number of dogs vaccinated was used as an estimation of doses applied. The number of doses applied may be larger than the number of dogs vaccinated in the case of biannual mass dog vaccination campaigns where a dog could be re-vaccinated in the same year. Doses applied was most consistently collected by PAHO and therefore used for this analysis.

For longitudinal REDIPRA data, missing data points were imputed using the last year of available data for each country and variable of interest. The figures display crude and imputed data separately.

During the analysis, several different categorizations were used to inform comparisons between the countries, including countries’ Gross National Income (GNI) per capita, population size and their sub-region (South America, Central America and Mexico, and the Caribbean). Of these, categorizing by GNI per capita was chosen by the authors for this analysis, as previous works have also shown the association between rabies incidence and country Gross Domestic Product [11]. Analyses were performed in R Version 3.2.2.

### Ethics statement

Annual human rabies case data aggregated at the country level was used for this paper. IRB approval was not sought for this data which is de-identified as case counts and publicly available through the SIRVERA website. The REDIPRA questionnaires do not contain patient data.

## Results

The data available from the REDIPRA surveys to evaluate the LAC countries rabies control programs is inconsistent (Table 1). The lowest participation rate was in 2006–2007 when only 10 countries provided data. The reason for this low participation rate is unknown. No data was collected for 2010–2011 due to a three-year gap, 2010 to 2013, between REDIPRA meetings, and the fact that for the 2013 REDIPRA meeting only data on 2012 was requested. No data was collected for 2010–2011. Current participation in the REDIPRA survey has reduced from the 1998–1999 levels.

**Table 1:** Number of countries that completed the REDIPRA questionnaires by main group of indicators by year. No PEP application data was collected for 2007. No data was collected for 2010–2011.

Year	Number of Countries Providing Data by Indicator		
	Dog Vaccine	Animal Exposures	PEP Application
1998	21	21	21
1999	21	21	20
2000	19	19	19
2001	19	19	20
2002	15	13	14
2003	13	13	14
2004	15	17	15
2005	15	17	16
2006	7	8	10
2007	7	8	0
2008	19	18	13
2009	18	19	21
2012	17	17	20
2013	16	17	19
2014	17	17	20

### Case and incidence data

**Fig 1:**
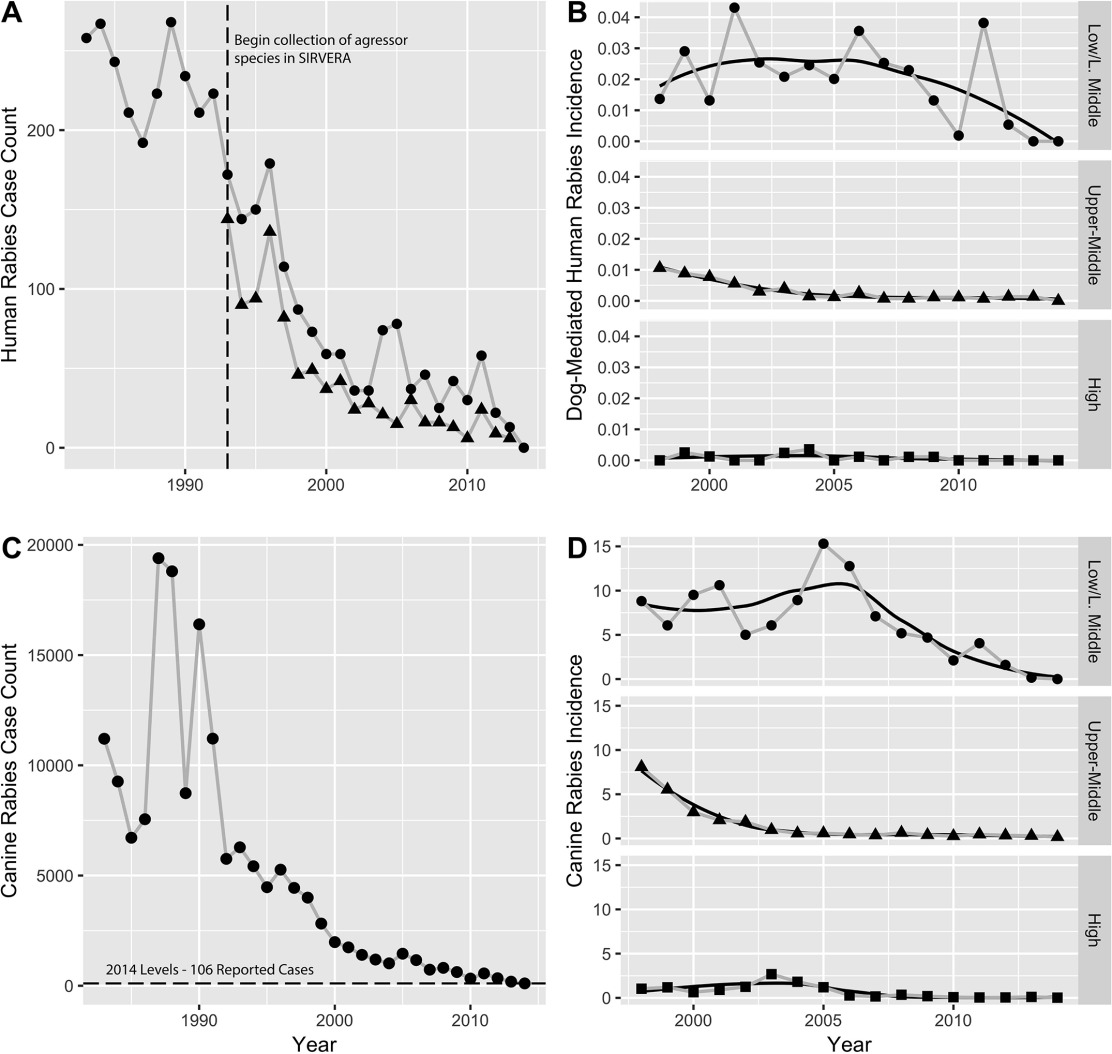
Human and dog rabies cases in LAC countries reported to SIRVERA. (A) Human case counts since the beginning of regional elimination program, period 1983–2014. Data collection on aggressor species began in 1993 (vertical dashed line). Human cases from all aggressor species are represented with circles from 1983 and dog-mediated cases with triangles from 1993 onward. (B) Dog-mediated human case incidence per 100,000 population by income category (WB, 2014) at the tail end of the epidemic with Loess smoother, period 1998–2014. (C) Dog case counts since the beginning of regional elimination program, period 1983–2014. (D) Dog rabies incidence per 100,000 dogs by income category at the tail end of the epidemic, period 1998–2014, using reported dog population.

Substantial declines in human rabies case counts in LAC countries have occurred since the 1980s (Fig 1A). The SIRVERA database began to collect data on aggressor species in 1993 allowing for the specific identification of dog-mediated human cases (Fig 1A). For the period covered by the REDIPRA surveys, 1998 to 2014, 778 human rabies cases were reported to SIRVERA in the 21 study countries, of which, 382 (49%) were dog mediated; bat-mediated rabies and unknown aggressor species were the second and third most reported with 298 cases (38%) and 44 (7%), respectively.

A consistent decline in was seen for dog rabies cases (Fig 1C). In the 17-year period (1998–2014), 20,442 dog rabies cases were reported to SIRVERA, an average of over 53 dog cases per each human case. In 2013 and 2014, 186 and 106 dog rabies cases were reported in the study countries, respectively.

Dog-mediated human rabies and dog rabies incidence rates by GNI per capita are shown in Fig 1B and 1C, respectively. High-income countries maintained low to zero incidence rates during the study period, while the upper middle-income countries followed a steady decline to 2005 with sporadic cases between 2005 and 2014. In the low and low-middle income countries the incidence rate was more variable and has not reached the steady, near zero rates found in the other income categories.

### Dog vaccination

**Fig 2:**
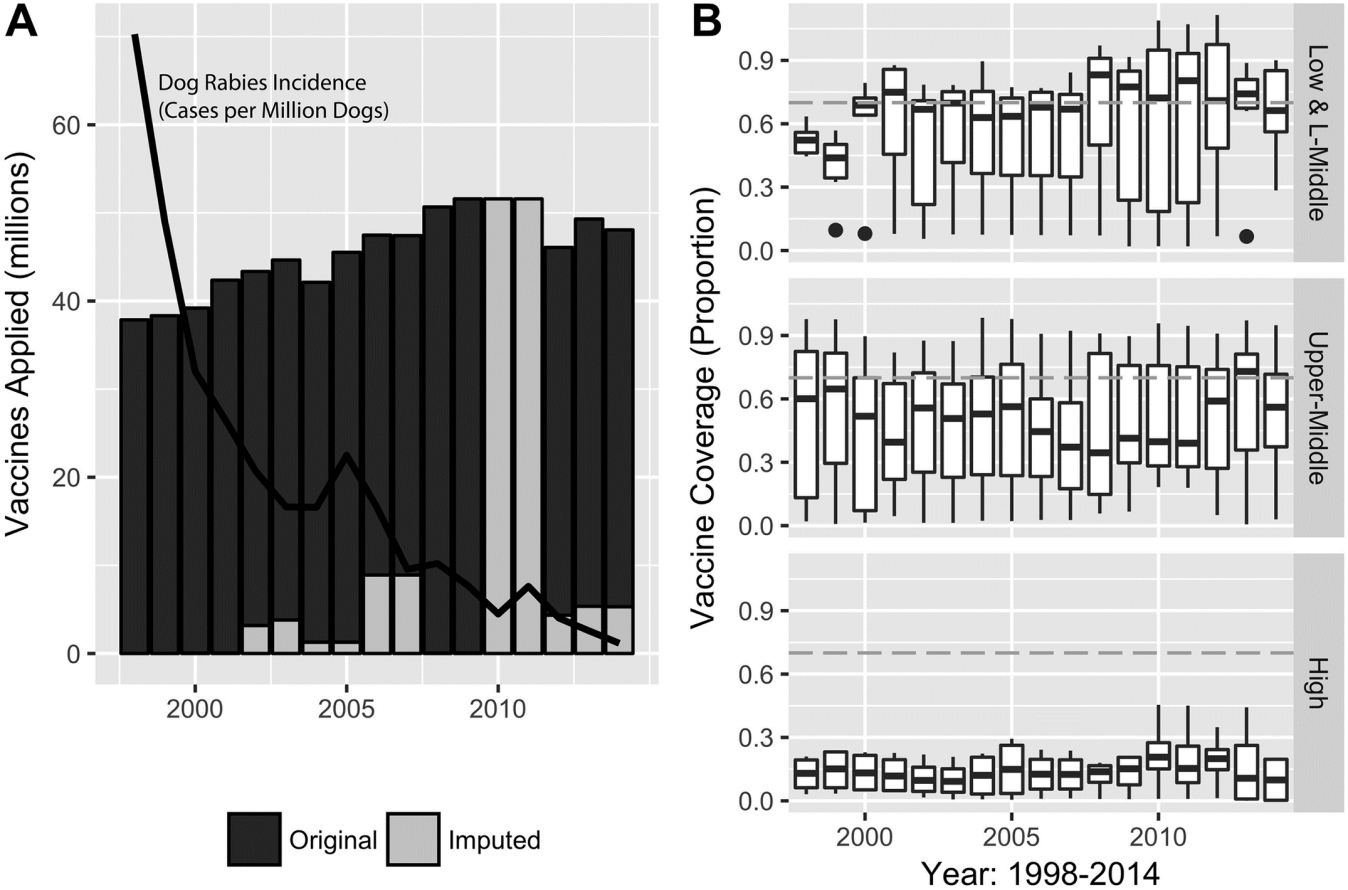
Dog vaccination in Latin America. (A) Number of Dogs Vaccinated in government campaigns per year in the region in the 21 reporting countries. Original Data is in black, imputed data is in grey. The incidence of dog rabies (per million dogs) in the region is overlaid in black for comparison (B) Box plots showing vaccination coverage rates for countries categorized by income category.

After accounting for missing data, the estimated number of dogs vaccinated in the 21 participating countries reached a peak in 2008–2009 with a decline in the overall number in the following years. The peak number of dogs vaccinated was an estimated 51.6 million dogs in 2009 (Fig 2A).

The estimated dog vaccination coverage rates varied greatly, both within countries across the study period and between countries. The coverage rates reported in this paper are based on estimated dog populations. No data was collected on the source of dog population estimates, but in the 2013–2014 REDIPRA questionnaire, 17 of 19 countries responded that dog populations were estimated using a dog: inhabitant ratio. The countries provided no information on how this ratio was derived. Two of 19 countries responded that a combination of methods was used to estimate dog population, including dog: inhabitant and animal census.

Vaccination numbers include government-purchased doses only and do not include private vaccination. High-income countries (n = 4), which report zero to sporadic dog rabies cases, have the lowest government-supplied vaccine coverage. The median coverage of the group of upper-middle income countries (n = 11) only once (in 2013) reached the recommended population immunity level of 70% (WHO, 2013) (Fig 2B).

Using 2013–2014 cost per dose as reported by the countries, the average unit cost of dog vaccine was $ 1.04 USD (Range: 0.15–2.95 USD) (Table 2). This estimate does not consider the full immunization costs, such as staff and supplies. In 2014, 7 countries purchased dog vaccine through PAHO’s Revolving Fund (a regional purchasing mechanism providing quality and competitive biologicals to the countries in the Region) at a price of 0.29 USD per dose, 6 purchased directly from private vaccine manufacturers, 7 produced the vaccine at national/public sector laboratories and 2 received donations from other countries in the region. Three countries reported more than one procurement mechanism. As of December 2014, three countries reported the continued use of NTV for dog vaccination. One of these countries used a mix of NTV and cell culture vaccine. Of the over 80 million doses applied in 2013–2014 in the 16 reporting countries, 5 million (6.2%) were NTV.

**Table 2:** Dog vaccine expenditure for years 2013–2014 showing the average unit cost per dose and type of vaccine (Nerve Tissue Vaccines (NTV) vs. cell culture) as reported by the countries, the number of doses applied, and the estimated total expenditure on vaccine (only vaccine, not including logistics costs) for 16 LAC countries.

	NTV (n = 3)		Cell Culture	
Average Dog Vaccine Price per dose 2013–2014 (USD)	$ 0.60 USD*		$ 1.04 (0.15–2.95 USD)	
Year	**2013**	**2014**	**2013**	**2014**
Number of Doses Applied	2,404,735	2,617,228	42,638,324	41,363,415
Total Estimated Cost of Dog Vaccine Doses Applied***	NA**	NA**	$28,907,054	$28,528,383

*Only one country supplied a unit cost for NTV.

**As limited data was available, the total cost was not estimated

*** SUM (Unit Cost Reported by country * Doses Applied)

PAHO Revolving Fund Pricing (0.29 USD per dose, 2014)

### Animal exposures and PEP

**Fig 3:**
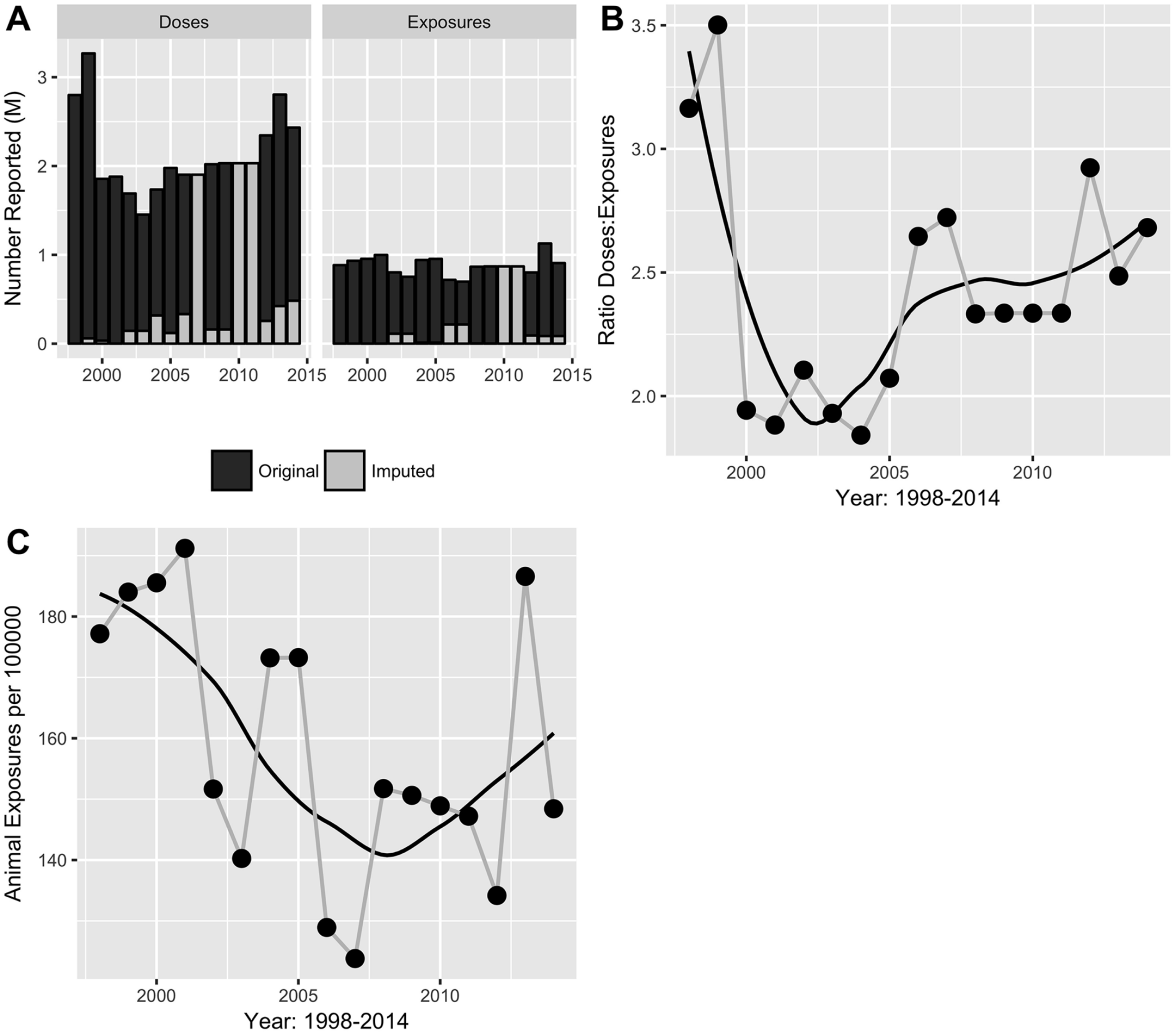
Animal exposures and post-exposure-prophylaxis (PEP). (A) Number of animal exposures and number of human rabies vaccines applied by year, period 1998–2014; (B) Ratio PEP doses to number of exposures; (C) Incidence of exposures per 100,000 population.

The number and incidence of animal exposures (includes all animals, information on dog-only was not available for most years) across the study countries was stable over time and trends did not vary greatly by income category (S1 Fig). The number of animal exposures reported annually ranged from 698,687 in 2007 to 1.1 million in 2013 in the study countries (Fig 3A). The range of animal exposure incidence fluctuated between a low of 124 per 100,000 in 2007 to a high of 191 per 100,000 in 2001 (Fig 3B).

As an indicator of PEP coverage, the ratio of PEP doses applied to exposures generally increased across time (Fig 3B and 3D). No data was available regionally on number of people receiving PEP, or number of people completing the PEP schedule. The exceptions to this trend were 1998 and 1999, where the highest number of PEP doses was applied (2.8 million and 3.3 million doses, respectively). This unusually high number was due solely to one country in the region, whose rates dropped ~50% in the subsequent years. It should also be noted that the 2014 numbers were lower than the 2013 numbers due primarily to incomplete vaccination data for the 2014 fiscal year for one country at the time of the REDIPRA survey.

In 2013 and 2014, over 2 million doses of human vaccine (cell culture and NTV) were applied annually in the 17 countries that responded to questions on human vaccines. Two countries reported the continued use of NTV with 188,305 doses applied over the two-year period. The total estimated PEP expenditure in the 17 responding countries was ~64 million USD over the two years. In 2013 and 2014, 131,659 and 107,392 doses of IG (both HRIG and ERIG) were applied in the 16 responding countries with an estimated expenditure of 6.9 million USD. Sixteen countries reported purchases from the PAHO Revolving Fund, 2 bought directly from private laboratories, 2 produced the vaccine at national/public sector laboratories and 2 received donations from other countries in the Region.

As of 2014, 16 of 17 responding countries recommend the Essen Method for PEP application (5 doses on days 0, 3, 7, 14 and 28) in their national guidelines. One country reported a similar schedule to the Essen (0, 3, 7, 14 and 30). Three countries, also recommend the reduced Essen scheme (4 Intramuscular doses on days 0, 3, 7 and 14); 3 countries also recommend the Zagreb Method (3 Intramuscular doses, 2 doses on day 0 and one dose on days 7 and 21); and 2 countries also recommend the Thai Red Cross scheme (2-site intradermal method on days 0, 3, 7 and 28).

### Surveillance and laboratory capacity

Of the 382 dog-mediated cases in the period 1998–2014, 76% were laboratory confirmed, 18.3% were clinically confirmed and 6% did not specify the diagnosis type. Data on PEP application in human cases is also collected in SIRVERA, but with a high rate of missing data. Of the 382 dog-mediated cases, 45 reported that the patient received at least one dose of vaccine, 155 (41%) reported that no vaccine was received and 182 (48%) did not provide this information. For IG, 21 (5%) received IG, 159 (42%) did not receive IG and 202 (48%) were unknown.

While SIRVERA started collecting data on negative surveillance samples in 2004, only nine of the study countries contributed information to the system. A total of 5,380 negative samples from these countries were reported over the 10-year period. Additional data on surveillance samples was collected by the 2013–2014 REDIPRA questionnaire. Seventeen of 19 countries responded, at least partially to the surveillance question. Two countries reported zero surveillance samples and zero cases and two countries did not respond to the total number of samples but did report positive cases. Over the two-year period, the 13 countries with at least one surveillance sample, sampled over 122,000 dogs for rabies surveillance (Table 3). Over 100,000 (83.3%) of the reported samples were contributed by one country alone. The positive rate was highest for countries that sampled a reduced proportion of their dog population (Table 3).

**Table 3:** Laboratory surveillance for years 2013–2014 from REDIPRA XV questionnaire. Of the 19 countries, 17 responded. Countries, labeled with a random numeric ID, are shown by income category.

ID	Dog Samples Submitted 2013–2014	Positive Dog Samples 2013–2014	Surveillance Samples as Percent of Total Dog Population	Percent of Surveillance Samples Positive for Rabies
Low and Low-Middle income				
7	119*	40*	0.01	33.61
8	246	12	0.01	4.88
12	251	1	0.01	0.40
13	298	0	0.02	0.00
3	Not Reported	48	_	_
Upper Middle Income				
2	0	0	0.00	_
6	661	2	0.01	0.30
9	289	50	0.01	17.30
14	13,138	32	0.02	0.24
15	1,794	20	0.03	1.11
16	1,578	48	0.07	3.04
17	101,889	21	0.30	0.02
High Income				
4	0	0	0.00	_
5	7	0	0.00	0.00
10	801	0	0.01	0.00
11	830	20	0.01	2.41
1	Not Reported	9	_	_

*Numbers available for 2014 only

For the same period, 17 countries responded to questions on in-country laboratory capacity: 14 (82.3%) reported the ability to perform Rabies diagnosis in the country (Table 4). Two responded that the type of laboratory testing available was unknown. Eight countries, four upper-middle and four high-income countries, reported that viral characterization of rabies was available in country. Five countries reported that serology was available. Of the four low-income countries responding, none reported the ability to perform viral characterization or serology in country.

**Table 4:** Rabies laboratory capacities. Percent of countries that replied positively to the use of various laboratory techniques for rabies diagnosis. Seventeen of 19 countries responded; the two non-responders included one low income and one upper middle income country.

Laboratory Capacity	Low and Low Middle Income (n = 4)		Upper Middle Income (n = 8)		High Income (n = 5)	
	n	%	n	%	n	%
**Rabies Diagnosis**	3	75%	6	75%	5	100%
Direct Fluorescent Antibody Test (DFA)	3	75%	5	63%	5	100%
Immunohistochemistry (IHC)	0	_	2	25%	1	20%
Viral RNA detection with RT-PCR	0	_	4	50%	2	40%
Histological examination of biopsy or autopsy tissues	0	_	3	38%	2	40%
Viral Isolation—Cell Culture	0	_	4	50%	0	_
Viral Isolation—Mice	2	50%	5	63%	5	100%
**Viral Characterization**	0	_	4	50%	4	80%
Genetic Characterization	0	_	4	50%	2	40%
Antigenic Characterization	0	_	4	50%	4	80%
**Serology**	0	_	4	50%	1	20%
Serology—Cell Culture	0	_	3	38%	1	20%
Serology—In Mice	0	_	4	50%	0	_
**Unknown**	1	25%	1	13%	0	_

### National program indicators

**Fig 4:**
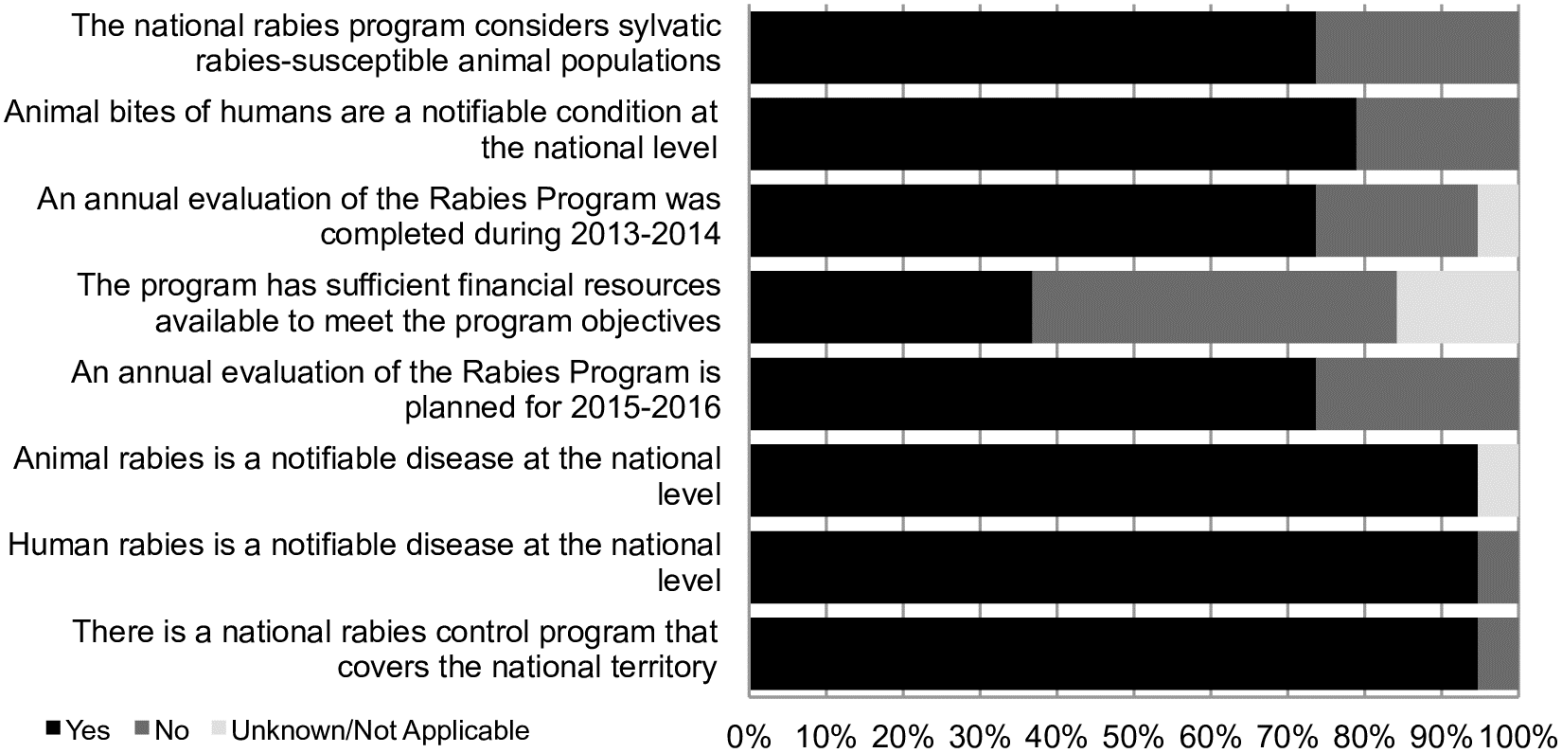
Indicators of national program capability in 2013–2014. Percentage of country respondents (n = 19) in 2013–2014 with national rabies control programs and basic indicators for control programs.

Limited data was available longitudinally on the status of the national rabies programs. For 2013 and 2014, 19 countries provided responses on their national program capabilities (Fig 4). All but one country had national rabies programs that cover the national territory. Human rabies is a reportable condition in all participating countries. Animal rabies is a reportable condition in all but one country. Animal exposures of humans are reportable in 15 of 19 countries (79%); of those countries where animal exposures are not a reportable condition, one was low income, one was upper middle and two were high-income countries. Notably, only seven (37%) of countries reported that they had sufficient financial resources to meet the program objectives. Three countries reported unknown on the question of financial resources. Of the nine countries that did not have sufficient financial resources to meet the program objectives, five were low and low middle-income countries (5 of 5), three were middle income (3 of 9) and one was a high-income country (1 of 5).

## Discussion

The collection of longitudinal data on a number of regional rabies indicators requires a substantial and concerted effort by a regional body. For the LAC region and rabies, PAHO has played this role over the last 34 years. Despite this coordinating effort, current country contribution to regional surveys has decreased from 1998 and 1999 levels. To encourage participation, it is important that the data, once collected, be analyzed and reported back to the countries. While rabies data is generally presented at the regular REDIPRA meetings every two years, the last published report of rabies programs indicators in LAC was in 2005 [12]. This paper summarizes the data collection efforts, focusing on the last 15 years and encourages continued participation in the regional rabies data collection efforts.

While regional dog-mediated rabies cases had decreased significantly by the start of the study period in 1998, there was still considerable variation in the endemicity status of the 21 study countries. We note that only 21 of the 47 PAHO Member States are described here, leading to the possibility of selection bias. The remaining countries were either free from dog-mediated rabies during the period of study (US, Canada, and most of the non-Spanish speaking countries in The Caribbean) or did not reply to our surveys or participate in SIRVERA throughout the years. In 1998, the Region had 46 reported human rabies cases and over 25,000 canine cases, while in the same year, four countries reached a goal of 5-years-free of dog-mediated human rabies (1993–97) (REDIPRA, 1998). This variation in rabies incidence can be seen throughout the study period and is reflected in the dog vaccination numbers, laboratory capacity, surveillance and national program indicators.

Sufficient and sustained dog vaccination campaigns underpin the regional success in rabies control. At its peak in 2008 and 2009, over 50 million doses per annum of government supplied dog rabies vaccine were applied throughout the Region. There was a possible reduction in government efforts from the 2008–2009 levels, which may be associated with the more sporadic occurrence of dog rabies observed in the Region. Some countries with zero dog rabies cases stopped providing mass dog vaccination campaigns during the study period, as evidenced by the low vaccine coverage rates in the high-income country group.

Despite the clear regional success, there still areas where rabies remains endemic. In contrast with the regional trend of sufficient and sustained dog vaccine coverage, these areas mostly reflect the opposite situation, one of lack of resources and logistics to mobilize the vaccine. Such local resource shortages most commonly stem from rabies programmes budget limitations, not only affecting the purchase of the vaccine but frequently the logistical arrangements required for vaccine deployment (e.g. vaccinators’ salaries, vehicles’ fuel). We stress at this point that mass dog vaccinations across the region, as well as the administration of PEP, are provided freely by the countries’ health systems. Despite this achievement, there are disparities in the availability of the biologicals, frequently reflecting deficient planning and/or the fragmented nature of sub-national purchasing mechanisms that fail to capitalize on economies of scale from bulk purchases.

Of the 21 countries in this study, at the time of writing (November 2016), only 1 country uses NTV for human rabies, and three countries for dog rabies. This number of countries has steadily declined over the study period. No recent information on the potency of the NTV used by the three countries, or of the occurrence of any adverse reactions, was available to the regional programme.

The number of animal exposures reported by the countries remained fairly stable during the study period and are likely to suffer from under-reporting, if only by comparison with the estimate for exposures in developing countries by Hampson et al of 239 exposures per 100,000 persons [1].

Given the elimination aspirations of the Region, the profile of the countries’ rabies programmes capacities is of great relevance (Fig 4). Critically, fewer than half of the respondents stated that their rabies control programmes had sufficient resources to sustain their activities. Importantly, the five low and low-middle income countries reported insufficient funds for their national programs. This information was only available for the last questionnaire of 2013–2014 and, hence, we cannot assess if this limitation was prevalent in earlier years. Other critical capacities, are those relating to monitoring and evaluation of the programmes’ performance [5]. Over 30% of the countries responded that they did not conduct evaluations of their programmes in 2013–14, and, worryingly, that they did not plan to do so for 2015–16.

Dog surveillance data was only available for 2013–14 and it shows a heterogeneous picture across the Region. Countries, by large, aimed to sample 0.01% or 0.02% of the dog population for surveillance purposes following existing dog surveillance recommendations [13]. Only one country exceeded this recommendation after testing most of the dogs culled and sterilized as part of the country’s dog population management programme. PAHO has commissioned further research to identify the most efficacious and cost-effective surveillance strategies for elimination of dog –mediated rabies in LAC [5].

The laboratory capacities data (Table 3) completes the landscape review of surveillance capability across the Region. Of note is the fact that fewer than half and just over one third of the countries reported they possessed the capacity of performing antigenic and genetic characterization, respectively. No low/low-middle income countries reported the capacity to perform viral characterization or serology in country. This is an acute deficiency given the need for targeted and precise approaches at the end game of disease elimination [5]. We also note the varying prevalence of the other laboratory capacities (Table 3), that captures, even if superficially, the large heterogeneity of laboratories practices as reported in greater detail in parallel studies [14].

### Limitations

While data is available at a national level, to protect the confidentiality of the countries, data was pooled and presented at a regional level or into income categories. Pooling the data shows regional longitudinal trends but obscures smaller in-country trends. In particular, large countries can have a visible impact on the results, such as the PEP numbers in 1998 and 1999, which were disproportionally contributed by one country.

A sensitive and statistically robust dog surveillance plays a critical role to inform progress in elimination [5]. While encouraged strongly through a variety of means, including at REDIPRA and during PAHO in-country missions, contribution of data to SIRVERA is voluntary. A number of countries, currently those still suffering from pockets of disease, have failed to systematically report to SIRVERA, and their responses to the pre-REDIPRA questionnaires were frequently incomplete. The incidence rates for rabies are based on the numbers reported to SIRVERA and therefore, the numbers presented may underestimate the overall burden of canine rabies in the region. To this end, we stress that dog rabies is underreported, as compared to human rabies, so the number of infected dogs for each human case is likely to be much greater than here reported, and so the size of the epidemic.

The estimated vaccine coverage rate is a crude estimate of the percent of the dog population receiving rabies vaccine. Calculated here as the number of doses applied over the estimated dog population, it does not take into account repeat vaccinations, nor does it consider the quality of the dog population estimates. No data was available on the methods used to estimate the dog populations reported in the REDIPRA reports prior to 2013. In the 2013–14 REDIPRA survey, the majority of the countries [17/19] reported using dog: inhabitant ratio to estimate dog population. The methodology for and the accuracy of dog population estimates remain a concern in the Region (REDIPRA 14). Further work towards standardizing dog population estimation was recommended during REDIPRA 14. Countries who consistently underestimated their dog populations and therefore, may undervaccinate their populations…

The reported vaccine costs do not include the full delivery cost of vaccination. The REDIPRA questionnaire for 2013–2014 asked for the unit cost per dose of dog vaccination and human PEP. Nevertheless, given the large spread of reported costs (0.15–2.95 USD), we cannot rule out that a few countries reported total delivery cost while others reported the unit cost of the biologic. The collection of additional cost data will prove useful to compare the cost-effectiveness of different vaccine delivery options for the varied geographic, cultural and endemicity scenarios in the Region.

Both SIRVERA and pre-REDIPRA questionnaires capture official figures mostly reported by Ministries of Health across the Region. The 2014 pre-REDIPRA questionnaire included data from both the Ministries of Health and of Agriculture to address this issue. Nevertheless, both systems fail to capture support from universities and private initiatives, e.g. vaccinations conducted by private veterinarians or PEP administered by private health care institutions, or those promoted and/or implemented by other institutions (e.g. NGOs) which may carry an important local impact.

This missing data from the historical REDIPRA records could introduce biases into the paper. The direction of the possible biases on the overall longitudinal trends is difficult to predict. For example, countries may have elected to participate only in successful vaccination years, or conversely, in difficult years, when regional assistance was required.

Other types of self-reporting biases are possible with this type of survey data. For example, the interpretation, definition and/or data collection of data points, such as vaccine doses applied, could vary by country. Some countries may include doses applied by non-governmental agencies in their overall dose count while others may include/exclude repeat vaccination of the same animal. Above all, the missing historical data highlights the need to consistently collect standardized data on the rabies effort.

The data for this study was pulled from historical paper records, prior to 2008 and while data entry was verified with two data entry persons and logic testing during data analysis, errors in either the historical record or in the current digitization are possible. Any errors due to historical or current data entry are likely to be random in nature.

### Opportunities

In general, and in particular for the end game of rabies elimination, standardization of monitoring and evaluation indicators is relevant to support data aggregation and comparisons with other areas for the identification of regional benchmarks. There is a need for the implementation of data quality checks to improve or complete data reported to SIRVERA and REDIPRA questionnaires. To our knowledge, there has not been a formal assessment of the quality of the data reported to SIRVERA. This is a required step in the maintenance and constant improvement of a disease notification database.

Improvements are possible in the form of regular reporting, and in the absence of cases, countries should report both surveillance results and rabies programs activities. A new SIRVERA 2.0 has been launched recently and aims to improve reporting by countries and the quality of their data by means of compulsory fields, a more comprehensive interface to query the database, and automatic email reminders [6].

In addition, a current effort by PAHO to support the rabies diagnostic laboratories in LAC countries, REDILAR (Rabies Laboratories Network), could be used to help improve surveillance data and encourage participation in SIRVERA. All these additions will increase the value of reporting to a regional database, but are not substitute for the footwork needed to convince country authorities to report in a sustained fashion. Advocacy by the regional program remains a priority.

### Conclusions

The sustained effort in LAC at the national and regional level over the last 4 decades produced a significant reduction in dog rabies incidence. PAHO has served as a consolidating force for the elimination effort and despite limitations, SIRVERA and the questionnaires from the biannual REDIPRA meetings are important regional tools for evaluating progress and refining the approach to control and elimination. Improvements in data collection are underway in the region, including a new SIRVERA platform capable of collecting surveillance and program capacity data. This study of the tail of the rabies epidemic in the LAC region demonstrates the need for endurance and continual improvement in the regional data collection, analysis, evaluation and communication efforts to support and inform the regional programme and the Member States.

## Supporting information

S1 Table De-identified country responses to the REDIPRA survey for 2013–2014. (PDF)

S1 Fig Reported animal aggression per 100,000 population by country GDP. (PDF)

S1 File Example of the REDIPRA questionnaire for REDIPRA 2012. (PDF)
