# Supplementary material for: Rabies in the Americas: 1998-2014
Source: PLoS Negl Trop Dis. 2018 Mar 20;12(3):e0006271. doi: 10.1371/journal.pntd.0006271 (PMC5877887; doi:10.1371/journal.pntd.0006271)
Supplement: S1 Table — (PDF) [file pntd.0006271.s001.pdf]

Supplemental Table 1: Responses to REDIPRA survey by Country for selected questions

Explanation of Codes. 1= YES, 0=No, 3=Unknown, Blank=Not Answered

**Country reported an exact 100:1 ratio. This was considered to be a data entry error and missing data. In 2012, the country reported a 10:1 ratio.*

Country Random ID	Human:Dog Population Raio		Human:dog ratio used to estimate the national dog population?	Country has an actively functioning electronic national registry system for Pre and Post Exposure Prophylaxis	Post-exposure vaccination scheme(s) are recommended by national guidelines					Current government purchasing mechanism(s) for HUMAN immunobiologics				Has a national system for purchasing and distributing canine / rabies vaccines	Current government purchasing mechanism(s) for CANINE vaccine				
	2013	2014			Essen Method- 5 Intramuscular doses (1-1-1-1-1) on days 0, 3, 7, 14 and 21 or 28	Reduced Essen Method - 4 Intramuscular doses (1-1-1-1-0) on days 0, 3, 7 and 14	Zagreb Method - 3 Intramuscular doses (2-0-1-0-1): 2 doses on day 0 and one dose on days 7 and 21	Thai Red-Cross Regiman - 2-site intradermal method (2-2-2-0-2) on days 0, 3, 7 and 28	Other	Purchased through Pan American Health Organization (PAHO) Revolving Fund	Purchased from private laboratories	Produced in national/public sector laboratories	Donated by other countries or organizations		Purchased through Pan American Health Organization (PAHO) Revolving Fund	Purchased from private laboratories	Produced in national/public sector laboratories	Donated by other countries or organizations	Other
107	9	9	1	1	1			1	1	1		1		1	1		1		
236	7	6	0	1	1				1		1			0		1			
434	100*	100*	1	0	3	3	3	3	3	1				1	1				
544	7	7	1	1	1					1		1		1	1		1		
771	8	7	1	0	1					1				1			1		
798	4	4	1	0	1					1				1		1			
1280	11	8	1	1	1					1				1		1			
1316	5	5	1	1		1	1		1	1				1	1				
1494	4	4	1	0	1					1				0		1			
1561	10	8	1	1	1		1			1				1			1		
1626	7	6	1	0	1	1				1				0					1
1641	Not Reported	8	1	0	1								1	1				1	
1689	5	7	1	1	1					1				1			1		
1697	5	5	1	1	1					1				1		1			
1802	11	11	0	1	1	1	1			1				1			1		
1855	8	8	1	0						1				1	1				
1882	8	8	1	0	1			1		1			1	1	1		1	1	
1903	10	10	1	0	1					1				1	1				
1973	5	5	1	1	1						1			1		1			

ued on Next Page

Country Random ID	Description of the national rabies program								Description of the rabies surveillance program			Tests available for use in national laboratories for the diagnosis or confirmation of rabies										
	There is a national rabies control program that covers the national territory	Human rabies is a notifiable disease at the national level	Animal rabies is a notifiable disease at the national level	An annual evaluation of the Rabies Program is planned for 2015-2016	The program has sufficient financial resources available to meet the program objectives	An annual evaluation of the Rabies Program was completed during 2013-2014	Animal bites of humans are a notifiable condition at the national level	The national rabies program considers sylvatic rabies-susceptible animal populations	Has an electronic surveillance system that aggregates HUMAN rabies case data at the national level	Has an electronic surveillance system that aggregates ANIMAL rabies case data at the national level	Has regular (at least annually) reporting at a national level of rabies surveillance data	Direct Fluorescent Antibody Test (DFA)	Viral RNA detection with RT-PCR	Histological examination of biopsy or autopsy tissues	Immunohistochemistry (IHC)	Virus isolation in cell cultures	Unknown	Viral Isolation in Mice	Antigenic Characterization	Genetic Characterization	Serology - Cell Culture	Serology - In Mice
107	1	1	1	1	2	1	1	1	1	1	1	1						1	1			
236	1	1	1	1	1	1	1	2	1	1	1	1						1	1	1		
434	1	1	1	1	1	1	1	1	1	3	1											1
544	1	1	1	1	1	1	1	2	1	1	1	1	1	1	1	1		1	1	1	1	1
771	1	1	1	2	2	2	1	1	1	1	1	1		1				1				
798	1	1	1	2	1	2	2	1	1	1	1	1	1					1	1	1		
1280	1	1	1	1	3	1	1	1	1	1	1	1	1			1						
1316	1	1	1	1	2	1	2	2	1		2											
1494	2	1	1	2	3	2	1	2	1	1	1	1		1	1			1	1			
1561	1	1	1	1	2	1	1	1	1	1	1	1	1	1		1		1	1	1	1	1
1626	1	1	1	1	1	3	2	1	2	2	1	1		1				1				
1641	1	1	1	2	2	2	1	1	1	1	1						1					
1689	1	1	1	2	2	1	1	2	1	1	1	1						1				
1697	1	1	1	1	2	1	1	1														
1802	1	1	1	1	2	1	1	1	1	1	1		1	1	1	1		1	1	1	1	1
1855	1	1	1	1	2	1	1	1	1	2	2	1										
1882	1	1	3	1	3	1	2	1	1	2	1	1						1				
1903	1	2	1	1	1	1	1	1	1	2	1						1					
1973	1	1	1	1	1	1	1	1	1	1	1	1	1					1	1	1	1	
