# Supplementary figures and images for: Rabies in the Americas: 1998-2014
Source: PLoS Negl Trop Dis. 2018 Mar 20;12(3):e0006271. doi: 10.1371/journal.pntd.0006271 (PMC5877887; doi:10.1371/journal.pntd.0006271)

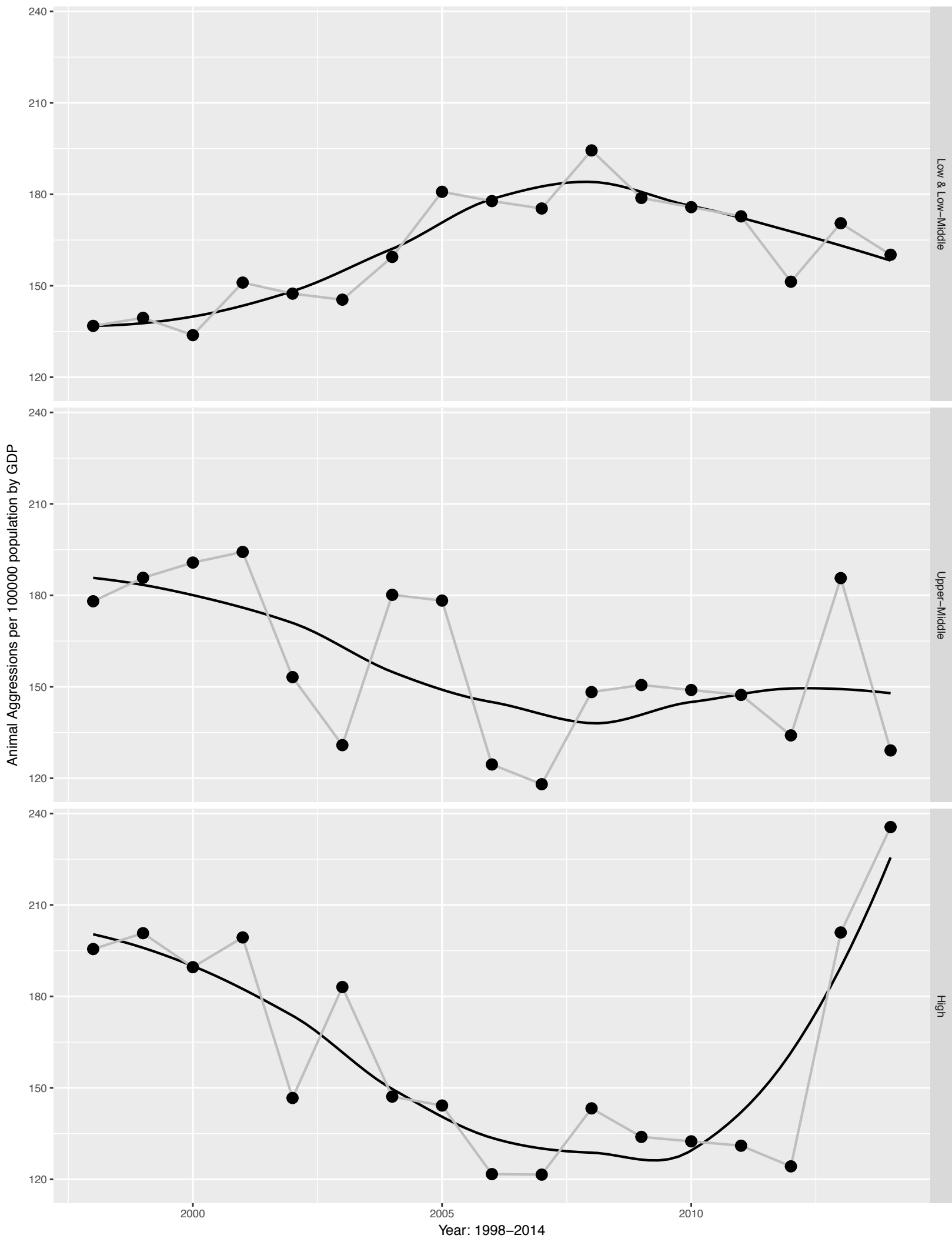

Supplement: S1 Fig — (PDF) [file pntd.0006271.s002.pdf]
