# Supplementary material for: Rabies in the Americas: 1998-2014
Source: PLoS Negl Trop Dis. 2018 Mar 20;12(3):e0006271. doi: 10.1371/journal.pntd.0006271 (PMC5877887; doi:10.1371/journal.pntd.0006271)
Supplement: S1 File — (PDF) [file pntd.0006271.s003.pdf]

Encuesta para la Reunión de Directores Nacionales de Rabia (REDIPRA). Año 2012

Estimados colegas

Es un placer poder dirigirnos a ustedes con motivo de la próxima reunión de directores nacionales de rabia (REDIPRA) que tendrá lugar en Lima, Perú, los días 20 al 22 de agosto de 2013. Este año la reunión está organizada conjuntamente por la Organización Panamericana de la Salud (OPS) y el Gobierno de Perú.

Para la preparación de la reunión, como en años anteriores, la OPS está recogiendo datos de los países participantes. A tal efecto, hemos preparado el cuestionario que se presenta más abajo. Las respuestas serán agregadas lo que permitirá obtener una imagen de la situación de la rabia en la región. La OPS presentará los resultados durante la conferencia en Lima. Le pedimos su colaboración respondiendo lo más exhaustivamente al cuestionario.

Instrucciones para completar el cuestionario (POR FAVOR LEA ATENTAMENTE)

El cuestionario se puede completar "on-line" y "off-line" (a través del fichero pdf anexado al email de introducción de la encuesta). En ambos casos, al final del cuestionario se pueden mandar las respuestas automáticamente a través del botón "Enviar" al final de la forma.

El cuestionario, en su preguntas 14 y 15, le requiere que se anexen el Plan Nacional de Rabia y su última evaluación respectivamente.
Igualmente, **si su pais tuvo casos de rabia humana, en perros o gatos, por cualquier variante, en el 2011 o 2012,** nos gustaría obtener el detalle regional. A tal efecto, le rogamos que complete las tablas relevantes del fichero Excel que acompañò al email de introducción de la encuesta. Estas tablas, una vez completadas se pueden anexar en la pregunta 40.

Desafortunadamente la forma en pdf no permite anexar ficheros. Dada esta limitación, le sugerimos los siguientes pasos para completar el cuestionario:

• Abra y complete off-line el cuestionario en su formato de pdf. La forma en pdf le permite guardar los cambios cuantas veces sea necesario. Una vez que el cuestionario en pdf este completo, por favor mándelo usando el botón "Enviar" al final de la forma. Por favor, asegúrese de que tiene conexión al Internet para el envio del fichero pdf.
• Una vez que haya mandado el pdf, abra la encuesta on-line. Rellene los campos obligatorios marcados con un asterisco. Vaya a la pregunta 14 y anexe su Plan Nacional de Rabia. Posteriormente vaya a la pregunta 15 y anexe la evaluación del Plan Nacional de Rabia.
• A continuación, solo si su pais tuvo casos de rabia en personas, perros o gatos en 2011 o 2012, vaya a la pregunta 40, y anexe el fichero de Excel con las tablas previamente completadas off-line, de acuerdo con la situación de rabia en su pais para esos dos años.
• Alternativamente a los dos pasos arriba detallados, usted puede hacer toda la operación on-line. Para ello le sugerimos que tenga toda la informacion preparada de antemano para rellenar el cuestionario. Note que el cuestionario on-line no salva cambios si decide dejar su sesión on-line.

Le rogamos que nos envie sus respuestas **antes del 19 de Julio.**

En caso de que tengan dudas o preguntas sobre el cuestionario, pueden mandarnos un email a vdelrio@paho.org

Muchas gracias por su colaboración

País (por favor, especifique su país)

Qué Ministerio representa?

☐ Ministerio/Departamento de Salud
☐ Ministerio/Departamento de Agricultura/Veterinaria
☐ Otro (por favor especificar)

Por favor note que el cuestionario cubre todo el programa de rabia, tanto en humanos como en animales. Por este motivo, algunas partes del mismo no se relacionarán directamente con las actividades de su Ministerio/Departamento. Por favor, ignórelas. Otras preguntas serán comunes a más de un departamento, por favor complételas con los datos directamente generados dentro su ministerio y red de vigilancia (por ejemplo, datos sobre murciélagos que en determinados países son recogidos por Ministerios de Salud (en núcleos urbanos) y Ministerios de Agricultura/Medio Ambiente (en zonas rurales)).

Datos del responsable de completar la encuesta (por favor note que estas preguntas son obligatorias. No podrá mandar sus datos si no se responden)

Nombre y apellidos

Datos de afiliación (nombre de la institución)

Departamento o grupo dentro de su institución

Cargo o posición

Teléfono (con código país y región)

Dirección de correo electrónico

Programa de rabia

1. Posee "Plan Nacional de Rabia" vigente?

☐ Sí ☐ No

2. Cual es el periodo de vigencia del Plan del plan actual (por ejemplo, del 2010 al 2015)?

3. Por favor, describa por que **NO** tiene "Plan Nacional de Rabia". Si lo tuvo en el pasado por favor describa su periodo de vigencia también (por ejemplo: del 2000 al 2005).

4. Seleccione la opción que mejor describa su Plan Nacional de Rabia (actual o pasado).

5. Por favor describa los objetivos principales de su Plan de Rabia (vigente o pasado) (por ejemplo, la declaración de área libre de rabia humana transmitida por perro en el territorio nacional para el 2015)?

6. Por favor, marque los mecanismos **formales** que sustentan (o sustentaron si su plan no es vigente) la integración de los planes de su Ministerio/Departamento contra la rabia con los de otros Ministerios/Departamentos. Marque todos los que correspondan

☐ Análisis integrado del riesgo de introducción de la rabia en cualquiera de sus formas

☐ Análisis integrado del riesgo de expansión de la rabia dentro del territorio nacional

☐ Estrategias y planes de investigación coordinados

☐ Intercambio sistemático y regular de datos epidemiológicos

☐ Comunicación regular y sistemática

☐ Evaluaciones regulares y sistemáticas de los programas de vigilancia y control

☐ Otros (especificar)

7. Cuál fue el coste (en US $) del programa de rabia en 2012 en su Ministerio/Departamento? Escriba "NA" si no conoce la respuesta

8. Del total del programa en 2012, cuanto correspondió (en US $) a vacunas para animales? Escriba "NA" si no conoce la respuesta

9. Del total del programa en 2012, cuanto correspondió (en US $) a inmunobiológicos para humanos? Escriba "NA" si no conoce la respuesta

10. Del total del programa en 2012, cuanto correspondió (en US$) a actividades de educación sanitaria. Escriba "NA" si no conoce la respuesta.

11. Del total del programa en 2012, cuanto correspondió (en US$) a actividades de investigación (básica, aplicada, investigación operacional). Escriba "NA" si no conoce la respuesta.

12. Del total del programa en 2012, cuanto correspondió (en US $) a actividades de vigilancia epidemiológica (incluyendo solo costes **directos** de campo, laboratorios, personal del Ministerio/Departamento en actividades de vigilancia pasiva y activa contra la rabia)

13. Cual ha sido la evolución del presupuesto de su Ministerio/Departamento para el control de la rabia en los ultimos 5 años (desde el 2008)? Por favor, proporcione las cantidades totales (US $) para cada año (por ejemplo: 2008 (1 millón US$), 2009 (800K US $), ...)

14. Por favor, anexe en el link abajo su Plan Nacional de Rabia vigente o el último que estuvo vigente.

Seleccionar archivo

15. Por favor, anexe en el link abajo la última evaluación de su Plan Nacional de Rabia

Seleccionar archivo

16. Por favor, explique aquí porque no ha podido anexar su Plan Nacional de Rabia, o la última evaluación del mismo, o cualquier otro aspecto del programa nacional de rabia en su país que considere oportuno mencionar.

Vigilancia epidemiológica

Vigilancia epidemiológica humanos

Año 2012. Datos nacionales

17. Número de laboratorios participantes en el diagnóstico de rabia humana en el país actualmente

18. Número de unidades de salud en todo el país con disponibilidad de inmunológicos para la rabia

19. Número de personas atacadas o expuestas a perros sospechosos de padecer rabia que fueron evaluadas por los servicios de salud de todo el país. Escriba "0" si ninguna

20. Número de personas atacadas o expuestas a perros sospechosos de padecer rabia a las que se les prescribió inmunoglobulina y vacuna. Escriba "0" si ninguna

21. Número de personas atacadas o expuestas a perros sospechosos de padecer rabia a las que solamente se les prescribió vacuna. Escriba "0" si ninguna

22. Del total de personas expuestas a perros sospechosos y que iniciaron tratamiento anti-rábico, cual fue el número de personas que terminaron el tratamiento?

23. Número de personas atacadas o expuestas a gatos sospechosos de padecer rabia que fueron evaluadas por los servicios de salud de todo el país. Escriba "0" si ninguna

24. Número de personas atacadas o expuestas a gatos sospechosos de padecer rabia a las que se les prescribió inmunoglobulina y vacuna. Escriba "0" si ninguna

25. Número de personas atacadas o expuestas a gatos sospechosos de padecer rabia a las que solamente se les prescribió vacuna. Escriba "0" si ninguna

26. Del total de personas expuestas a gatos sospechosos y que iniciaron tratamiento anti-rábico, cual fue el número de personas que terminaron el tratamiento?

27. Número de personas atacadas o expuestas a murciélagos que fueron evaluadas por los servicios de salud de todo el país. Escriba "0" si ninguna

28. Número de personas atacadas o expuestas a murciélagos a las que se les prescribió inmunoglobulina y vacuna. Escriba "0" si ninguna

29. Número de personas atacadas o expuestas a murciélagos a las que solamente se les prescribió vacuna. Escriba "0" si ninguna

30. Del total de personas expuestas a murciélagos y que iniciaron tratamiento anti-rábico, cual fue el número de personas que terminaron el tratamiento?

31. Número de personas atacadas o expuestas a animales silvestres (e.g. zorros) que fueron evaluadas por los servicios de salud de todo el país. Escriba "0" si ninguna

32. Número de personas atacadas o expuestas a animales silvestres (e.g. zorros) a las que se les prescribió inmunoglobulina y vacuna. Escriba "0" si ninguna

33. Número de personas atacadas o expuestas a animales silvestres (e.g. zorros) a las que solamente se les prescribió vacuna. Escriba "0" si ninguna

34. Del total de personas expuestas a animales silvestres (e.g. zorro) y que iniciaron tratamiento anti-rábico, cual fue el número de personas que terminaron el tratamiento?

35. Número de personas atacadas o expuestas a animales de producción (vacuno, equino, etc) sospechosos de padecer rabia que fueron evaluadas por los servicios de salud de todo el país. Escriba "0" si ninguna

36. Número de personas atacadas o expuestas a animales de producción (vacuno, equino, etc) sospechosos de padecer rabia a las que se les prescribió inmunoglobulina y vacuna. Escriba "0" si ninguna

37. Número de personas atacadas o expuestas a animales de producción (vacuno, equino, etc) sospechosos de padecer rabia a las que solamente se les prescribió vacuna. Escriba "0" si ninguna

38. Del total de personas expuestas a animales de producción sospechosos y que iniciaron tratamiento anti-rábico, cual fue el número de personas que terminaron el tratamiento?

39. Número de pacientes que fueron testados contra la rabia, por cualquier exposición, en todo el país.

---

40. SI en el 2011 o 2012 tuvo casos humanos, en perros o en gatos, por cualquier variante, nos interesa saber sobre el detalle regional. A tal efecto, por favor complete las tablas que se relacionan a continuación y que forman parte del fichero Excel que acompañó al email de introducción del cuestionario. Una vez que complete las tablas que le correspondan con la información pertinente, por favor anexe el fichero Excel (usando el botón "Seleccionar archivo"). Por ejemplo, si su país tuvo casos humanos, en perros y en gatos en 2011 y 2012, debiera completar las siete tablas. Si, por ejemplo, sólo tuvo casos humanos en 2012 y en perros en 2011, debiera completar las Tablas "Casos_humanos_PPE_2012", "Exposición_PPE_2012" y "Perros_vigilancia_2011".

1. Casos humanos en 2011: "Tabla 1: Casos_humanos_PPE_2011" y "Tabla 3: Exposición_PPE_2011"
2. Casos humanos en 2012: "Tabla 2: Casos_humanos_PPE_2012" y "Tabla 4: Exposición_PPE_2012"
3. Casos en perros en 2011: "Perros_vigilancia_2011".
4. Casos en perros en 2012: "Perros_vigilancia_2012"
5. Casos en gatos en 2011 o 2012: "Gatos_vigilancia"

Seleccionar archivo

Vigilancia epidemiológica animales

41. Por favor, seleccione todas las opciones que se encuentren dentro de las competencias de su Ministerio/Departamento en relación a la vigilancia epidemiológica de la rabia.

- ☐ Animales de abasto en zonas rurales y urbanas
- ☐ Animales silvestres (por ejemplo murciélagos, zorros) en áreas rurales
- ☐ Animales silvestres (por ejemplo murciélagos, zorros) en áreas urbanas
- ☐ Perros y gatos (animales domésticos) en áreas rurales
- ☐ Perros y gatos (animales domésticos) en áreas urbanas
- ☐ Otras (especificar)

42. Número de laboratorios participantes en el diagnóstico de rabia animal en el país, actualmente, dependientes directamente de su Ministerio/Departamento

43. Número de laboratorios participantes en el diagnóstico de rabia animal dependientes de otros Departamentos/Ministerios. Escriba "0" si ninguno. Escriba "UNK" si lo desconoce.

44. Es la rabia en animales una enfermedad de notificación obligatoria?

- ☐ Sí
- ☐ No
- ☐ Otros (especificar)

Perros.

45. Ultimo estimado **Nacional** de la población canina (e.g. 2325000).

46. A que año corresponde este estimado de la población canina

47. Por favor, describa los métodos de estimación de las poblaciones caninas recomendados por su Ministerio/Departamento.

48. Condujo **vigilancia activa** de rabia en perros en 2012?

☐ Sí, en todo el territorio nacional

☐ No

☐ Sólo en áreas específicas (especifique, por ejemplo, áreas de frontera, endémicas)

49. Por favor describa **todas** las fuentes de los perros testados dentro de la vigilancia activa (por ejemplo, animales sacrificados en caníles públicos, en caniles privados, etc). Escriba "NA" si no condujo vigilancia activa en el 2012

50. Los perros testados dentro de la vigilancia activa eran? (ignore si no condujo vigilancia activa en el 2012)

	Nunca (0%)	Escasamente (25%)	En la mitad de los casos (50%)	A menudo (75%)	Siempre (100%)
Perros con sintomatología nerviosa	☐	☐	☐	☐	☐

51. Por favor, describa que acciones **locales** (**de vigilancia y control**) se describen en sus procedimientos ante el diagnóstico de rabia en perro (por ejemplo, vacunación de bloqueo)

52. Por favor describa el tamaño del área (su radio en Km) donde se recomienda vacunación de emergencia una vez que se encuentra un caso positivo de rabia en perro. Describa que criterios (densidad poblacional, etc) aplica si este radio varia, y cual es el objetivo de cobertura vacunal (en % de la población censada).

Año 2012. Datos nacionales (sólo perros)

53. Vigilancia pasiva. Número de perros **sospechosos** de rabia en todo el país. Escriba "0" si ninguno

54. Vigilancia pasiva. Número de perros **sospechosos testados** por rabia en todo el país. Escriba "0" si ninguno.

55. Vigilancia activa. Número de perros requerido en todo el pais (este total se puede derivar de la suma de diversas regiones) para alcanzar el poder muestral requerido por la vigilancia activa. Escriba "NA" si no condujo vigilancia activa

56. Vigilancia activa. Que proporción de la población de perros en zonas de riesgo suponía el número de perros requerido en la pregunta anterior (por ejemplo, 0.1%)?

57. Vigilancia activa. Número de perros testados en todo el pais, solo dentro de la vigilancia activa. Escriba "NA" si no condujo vigilancia activa

Gatos

58. Ultimo estimado **Nacional** de la población felina (1230000)

59. A que año corresponde este estimado de la población felina

60. Por favor, describa los métodos de estimación de las poblaciones felinas recomendados por su Ministerio y que son en su mayoría seguidos por las administraciones locales. Si son las mismas que para perros, sólo escriba "Igual que en perros"

Año 2012. Datos nacionales.

61. Vigilancia pasiva. Número de gatos **sospechosos** de rabia en todo el país. Escriba "0" si ninguno

62. Vigilancia pasiva. Número de gatos **sospechosos testados** por rabia en todo el país. Escriba "0" si ninguno

Murciélagos

63. Describa que tipo de actividades de vigilancia activa de rabia en murciélagos desarrolla de manera rutinaria su Ministerio/ Departamento. Escriba "NA" si ninguna.

64. Por favor, describa que acciones **locales** (**de vigilancia y control**) se describen en sus procedimientos, especificos de su Ministerio/Departamento, ante el diagnóstico de rabia en murciélago (por ejemplo, vacunación de bloqueo en perros y gatos)

Año 2012. Datos nacionales

65. Número de murciélagos, de cualquier especie, testados por rabia en todo el territorio nacional

66. Número de murciélagos, de cualquier especie, confirmados con rabia, de cualquier variante, en todo el territorio nacional.

67. Número de murciélagos confirmados con rabia de variante 3. Escriba "0" si ninguna. Escriba "NA" si no tiene datos.

68. Número de murciélagos confirmados con rabia de otras variantes (4, 5, 6). Escriba "0" si ninguna. Escriba "NA" si no tiene datos.

69. Del total de murciélagos positivos, por cualquier variante, recogidos directamente por la red de vigilancia de su Ministerio/ Departamento, cuantos fueron recogidos en zonas urbanas?

70. Del total de murciélagos positivos, por cualquier variante, recogidos directamente por la red de vigilancia de su Ministerio/ Departamento, cuantos fueron recogidos en zonas peri-urbanas?

71. Del total de murciélagos positivos, por cualquier variante, recogidos directamente por la red de vigilancia de su Ministerio/ Departamento, cuantos fueron hematófagos?. Escriba "0" si ninguno. Escriba "NA" si no hay datos.

72. Del total de murciélagos positivos, por cualquier variante, recogidos directamente por la red de vigilancia de su Ministerio/ Departamento, cuantos fueron no-hematófagos? Escriba "0" si ninguno. Escriba "NA" si no hay datos.

73. Por favor, proporcione la distribución de murciélagos positivos recogidos directamente por su Ministerio/Departamento, por especie (por ejemplo, Molossus molossus (n=3), Molossus musculus (n=2), etc). Si no tuviera el detalle por especies, por favor proporcione por genero (Molossus) o Familia (Molossidae, Phyllostomidae, etc). Escriba "0" si ninguno. Escriba "NA" si no tiene datos.

Herbívoros

Año 2012. Datos nacionales

74. Número de animales de producción de cualquier especie testados por rabia en todo el territorio nacional. Por favor proporcione detalle de las especies (por ejemplo: bovino (1000 casos), equino (500 casos)). Escriba "0" si ninguno. Escriba "NA" si no tiene datos

75. Número de animales de producción, de cualquier especie, confirmados con rabia, de cualquier variante, en todo el territorio nacional. Por favor proporcione detalle de las especies (por ejemplo: bovino (800 casos), equino (400 casos)). Escriba "0" si ninguno. Escriba "NA" si no tiene datos

---

Animales salvajes/fauna silvestre (no murciélagos)

Año 2012. Datos nacionales

76. Número de animales salvajes de cualquier especie testados por rabia en todo el territorio nacional. Por favor proporcione detalle de las especies (por ejemplo: zorro (100 casos), mono (50 casos)). Escriba "0" si ninguno. Escriba "NA" si no tiene datos

77. Número de animales salvajes, de cualquier especie, confirmados con rabia, de cualquier variante, en todo el territorio nacional. Por favor proporcione detalle de las especies (por ejemplo: zorro (20 casos), mono (4 casos)). Escriba "0" si ninguno. Escriba "NA" si no tiene datos

---

Técnicas diagnósticas

Introduzca el número total de tests empleados para TODAS las muestras procesadas por TODA la red de laboratorios dependiente de su Ministerio/Departamento. Marque "0" si el test no fue utilizado. Marque "x" si el test fue utilizado pero desconoce el número de tests usados.

Año 2012. Datos nacionales

78. Número de pruebas de inmunofluorescencia directa (IFD)

79. Número de pruebas de aislamiento en ratones lactantes

80. Número de pruebas de aislamiento en cultivo celular

81. Número de pruebas RT-PCR

82. Número caracterización viral con monoclonales

83. Número caracterización viral por secuenciamiento ADN

84. Número seroneutralización en ratones

85. Número seroneutralización en células con técnica de inhibición de focos fluorescentes RIFFT

---

86. Manual de procedimientos técnicos para el diagnóstico de rabia implementado a nivel nacional. Marque todos los que corresponda.

- ☐ Sí
- ☐ No
- ☐ Sólo algunos laboratorios
- ☐ Otro (especificar)

87. Manual de bioseguridad implementado en los laboratorios que manipulan rabia de la red de laboratorios de su Ministerio/ Dpto.

- ☐ Sí
- ☐ No
- ☐ Sólo algunos laboratorios
- ☐ Otro (especificar)

88. Manual de gestión de calidad implementado en los laboratorios que manipulan rabia de la red de laboratorios de su Ministerio/Dpto

- ☐ Sí
- ☐ No
- ☐ Sólo algunos laboratorios
- ☐ Otro (especificar)

Profilaxis anti-rábica e inmunobiológicos

Personas

Año 2012. Datos nacionales

89. Número de vacunas **disponibles** para uso humano (de cerebro de ratón lactante) producidas por Laboratorios Nacionales Públicos. Escriba "0" si ninguna

90. Número de vacunas **disponibles** para uso humano (de cerebro de ratón lactante) recibidas en donación por otros países. Por favor detalle el país y el número de vacunas (por ejemplo: País A 5000 vacunas). Escriba "0" si ninguna

91. Número de vacunas **disponibles** para uso humano (en cultivo celular) producidas por Laboratorios Nacionales Públicos. Escribir "0" si ninguna.

92. Número de vacunas **disponibles** para uso humano (en cultivo celular) compradas a laboratorio privados. Por favor, detalle el nombre del laboratorio y el número de vacunas compradas (por ejemplo: Laboratorio X 10000 vacunas). Escribir "0" si ninguna

93. Número de vacunas **disponibles** para uso humano (en cultivo celular) recibidas en donación por otros países. Por favor detalle el país y el número de vacunas (por ejemplo: País A 5000 vacunas). Escriba "0" si ninguna

94. Si donó vacunas a otros países, por favor especifique los países, el tipo de vacuna y la cantidad (de la siguiente forma: País "x" 1000 vacunas de cultivo celular, Pais "y" 6000 vacunas de cultivo celular)

95. Número de vacunas de cerebro de ratón lactante **aplicadas** para uso humano. Escriba "0" si ninguna

96. Número de reacciones adversas a la aplicación de vacuna de ratón lactante. Escribir "0" si ninguna. Escriba "NA" si no tiene datos

97. Número de vacunas en cultivo celular **aplicadas** para uso humano. Escriba "0" si ninguna.

98. Número de reacciones adversas a la aplicación de vacuna en cultivo celular. Escribir "0" si ninguna. Escriba "NA" si no tiene datos

99. Por favor, describa brevemente el número de vacunas desechadas, de cualquier tipo, durante el 2012 por cualquier causa (expiración, rotura cadena de frio, etc). Escriba "NA" si no tiene datos

100. Por favor, comente aquí cualquier incidencia o éxito del programa de vacunación en el 2012.

Año 2013. Datos nacionales

101. Por favor, proporcione el número de vacunas (de cerebro de ratón lactante) planificadas para su uso en 2013. Escriba "0" si ninguna

102. Por favor, proporcione el número de vacunas (en cultivo celular) planificadas para su uso en 2013. Escriba "0" si ninguna.

Perros y gatos

103. Es la vacunación **de rutina** de perros, **a día de hoy**, obligatoria en todo el terreno nacional?

☐ No ☐ Sí

☐ Otro (por ejemplo, sólo en áreas endémicas)

104. Son campañas anuales?

☐ Sí ☐ No

105. Es la vacunación **de rutina** de gatos, **a día de hoy**, obligatoria en todo el terreno nacional?

☐ No ☐ Sí

☐ Otro (por ejemplo, sólo en áreas endémicas)

106. Son campañas anuales?

☐ Sí ☐ No

Año 2012. Datos nacionales

107. Por favor, proporcione el número de perros que se planeó vacunar en la campaña de vacunación del 2012 en todo el pais

108. Número de perros que se vacunaron en todo el territorio nacional en el 2012?

109. Por favor, proporcione el número de gatos que se planeó vacunar en la campaña de vacunación del 2012 en todo el pais

110. Número de gatos que se vacunaron en todo el territorio nacional en el 2012?

111. Número de vacunas **disponibles** para uso en perros y gatos (de cerebro de ratón lactante) producidas por Laboratorios Nacionales Públicos. Escriba "0" si ninguna

112. Número de vacunas **disponibles** para uso en perros y gatos (de cerebro de ratón lactante) recibidas en donación por otros países. Por favor detalle el país y el número de vacunas (por ejemplo: País A 5000 vacunas). Escriba "0" si ninguna

113. Número de vacunas **disponibles** para uso en perros y gatos (en cultivo celular) producidas por Laboratorios Nacionales Públicos. Escribir "0" si ninguna.

114. Número de vacunas **disponibles** para uso en perros y gatos (en cultivo celular) compradas a laboratorio privados. Por favor, detalle el nombre del laboratorio y el número de vacunas compradas (por ejemplo: Laboratorio X 10000 vacunas). Escribir "0" si ninguna

115. Número de vacunas **disponibles** para uso en perros y gatos (en cultivo celular) recibidas en donación por otros países. Por favor detalle el país y el número de vacunas (por ejemplo: País A 5000 vacunas). Escriba "0" si ninguna

116. Si donó vacunas a otros países, por favor especifique los países, el tipo de vacuna y la cantidad (de la siguiente forma: País "x" 1000 vacunas de cultivo celular, Pais "y" 6000 vacunas de cultivo celular)

117. Número de vacunas de cerebro de ratón lactante **aplicadas** para uso en perros y gatos . Escriba "0" si ninguna

118. Número de reacciones adversas a la aplicación de vacuna de ratón lactante. Escribir "0" si ninguna. Escriba "NA" si no tiene datos

119. Número de vacunas en cultivo celular **aplicadas** para uso en perros y gatos. Escriba "0" si ninguna.

120. Número de reacciones adversas a la aplicación de vacuna en cultivo celular. Escribir "0" si ninguna. Escriba "NA" si no tiene datos

121. Por favor, describa brevemente el número de vacunas desechadas, de cualquier tipo, durante el 2012 por cualquier causa (expiración, rotura cadena de frio, etc). Escriba "NA" si no tiene datos

122. Por favor, comente aquí cualquier incidencia o éxito del programa de vacunación en el 2012.

---

123. Por favor describa como su Ministerio/Departamento evalúa el grado de concientización del riesgo de rabia, por cualquier variante, entre la población. Escriba "NA" si no tiene datos. Describa la frecuencia de estas evaluaciones.

124. Que habría que mejorar de manera prioritaria en su país para el manejo (prevención, detección e intervención) **eficiente** de la rabia humana transmitida por perro? (Aplica tanto a países con casos de rabia transmitida por perro como aquellos que la han eliminado (en este caso, considere el riesgo). Por favor, describa desde la perspectiva de su Ministerio/Departamento.

125. Cual es la necesidad más acuciante en su país para el manejo completo (prevención, detección e intervención) de la rabia silvestre?. Por favor, describa desde la perspectiva de su Ministerio/Departamento

126. Que iniciativas de tipo técnico cree usted que son necesarias para la eliminación de la rabia humana transmitida por perro en la **región** de las Americas para el 2015.

127. Que iniciativas de tipo político cree usted que son necesarias para la eliminación de la rabia humana transmitida por perro en la **región** de las Américas para el 2015.

128. Cómo contribuiría mejor PAHO a las necesidades del país en cuanto a control de rabia en general? Por favor describa.

Muchas gracias por su tiempo. Sus respuestas son importantes para la actualización de la situación de la rabia en el continente. Por favor, revise una vez más el cuestionario y responda a todas las preguntas.

Atentamente

El grupo de zoonosis de la Organización Panamericana de la Salud.

Enviar
